# In vitro characterization of osteoblasts from craniofacial fibrous dysplasia of bone and their impact on bone homeostasis

**DOI:** 10.1186/s13023-026-04262-0

**Published:** 2026-02-24

**Authors:** Johan Sergheraert, Claire Dumortier, Christine Guillaume, Sébastien Laurence, Fabien Bornert, Sophie C. Gangloff, Cédric Mauprivez, Frédéric Velard

**Affiliations:** 1 Université de Reims Champagne Ardenne, BIOS, Reims, France; 2 Centre Hospitalo-Universitaire de Reims, Pôle de Médecine Bucco- Dentaire, Reims, France; 3 Université de Reims Champagne Ardenne, UFR d’Odontologie, Reims, France; 4 Hôpitaux Universitaires de Strasbourg, Pôle de Médecine et de Chirurgie Bucco-Dentaires, Strasbourg, France; 5 Université de Reims Champagne Ardenne, UFR de Pharmacie, Reims, France

**Keywords:** Fibrous dysplasia of bone, Primary human osteoblasts, Osteoblast differentiation, Primary human monocytes, Osteoclastogenesis

## Abstract

Fibrous Dysplasia (FD) is a benign and rare genetic disease characterized by progressive replacement of normal bone by a non-mineralized matrix. FD is caused by GNAS mutations that increase intracellular cAMP levels. While appendicular lesions have been extensively studied to enhance understanding and management of the disease, the pathogenesis of craniofacial lesions remains poorly described despite the embryological, phenotypic and mechanical differences between these two skeletal regions. Our objective was to evaluate the impact of craniofacial FD on bone homeostasis. Typical histological features, suh as “alphabet soup” appearance, were observed in craniofacial FD lesions. Higher intracellular cAMP levels were also noted in FD cultures derived from craniofacial lesions. We demonstrated an increased proliferative capacity in craniofacial FD osteoblasts, associated with decreased osteoblastic differentiation and a lack in mineralization abilities. Interestingly, we highlighted a decreased osteoclastogenic potential due to impaired osteoclast formation in FD-conditioned medium cultures, indicating a diminished osteoclastogenic potential of craniofacial FD osteoblasts. Our data suggest shared characteristics between osteoblasts from the craniofacial and the appendicular skeletons but also discrepancies regarding osteoclastic potential. Exploring these features could help explain the differences observed between appendicular and cranio-facial lesions and should lead to further studies on the pathogenesis of craniofacial fibrous dysplasia.

**Supplementary Information:**

The online version contains supplementary material available at 10.1186/s13023-026-04262-0.

## Introduction

Fibrous Dysplasia of bone (FD)(ICD-O 8818/0) is a rare benign mosaic disease affecting the skeleton [1–3]. FD is caused by a post-zygotic somatic activating mutation in the *GNAS* gene (*Guanine nucleotide binding protein, alpha stimulating*). These mutations lead to constitutive activation of adenylyl cyclase and elevated levels of intracellular cyclic AMP. This congenital and non-hereditary disease is characterized by a progressive replacement of normal bone with fibro osseous lesions [4–7].

FD is classified as a skeletal disorder and characterized by unifocal or multifocal expanding fibrous lesions inside the bone tissue. FD is a rare disease without sexual predominance [3, 8, 9]. FD can be in isolation or as part of syndromic forms associated with extraskeletal features including endocrine abnormalities (precocious puberty, hyperthyroidism, growth hormone excess, renal phosphate excess, …) leading to the diagnosis of McCune-Albright syndrome (MAS) [10].

Craniofacial involvement is reported in 25% of cases with monostotic FD [11] and about 10% of all FD cases [12]. Both monostotic, the most frequent one, and polyostotic FD share similar radiological and histological features. Radiographically, the fibro-osseous lesions are typically well defined with a ground-glass appearance. Less commonly, the lesions are either radiolucent (cystic appearance) or more radiopaque (sclerotic appearance). Histologically, the normal bone tissue is replaced by irregular trabeculae of immature bone within in cellular fibrous stroma.

Clinical manifestations or symptoms leading to FD diagnosis are pain due to nervous compression and skeletal distortions which can result in musculoskeletal disabilities and aesthetic issues [13–15]. In the cephalic area, frontal and occipital bumps, as well as prognathism, are the most common manifestations of hypertrophic lesions. Dental findings and sensory abnormalities may also aid in FD diagnosis [16]. Current treatments are limited to symptomatic management and based on pharmacological and surgical therapeutics [17–19]. Nowadays, antiresorptive agents (bisphosphonate [20] and Denosumab, a RANK-L inhibiting therapy [21]) are considered to reduce pain although results vary. Resective surgery remains the most common treatment approach [20, 22].

 In vitro and in vivo studies have highlighted the presence of both FD (mutant) and non-FD (wild-type) cells in affected tissue. Mutants cells exhibit higher proliferative capabilities compared to WT cells from the appendicular skeleton [4]. Histological analysis reveals decreased osteoblastic differentiation that highlights immature osteoid tissue associated with extensive fibrosis [23]. Early markers of osteoblastic differentiation (RUNX2, osteonectin, osteopontin) have not been impacted by GNAS mutation. Furthermore, two late markers, namely osteocalcin and bone sialoprotein, were weakly expressed or absent [24–26]. Histological data have evidenced a mineralization defect with increased surface of non-mineralized tissue and increased mineralization delay [4]. In vitro studies on appendicular skeleton cells have shown increased secretion of IL-6 and IL-11 [27, 28], two pro-osteoclastogenic factors that modulate bone homeostasis and osteoclast differentiation [27–29]. Recently, Michel et al. observed an increase of pro-inflammatory cytokines as IL-2, -4, -7, -8 and TNFα in supernatants from mice bearing mutation R201C [30]. However, IL-6 is also described as an anti-osteoclastogenic cytokine involved in bone remodeling, suppressing RANK (receptor activator of NF-κB) signaling pathways on osteoclasts precursors [31, 32]. The effects of IL-6 on osteoclastogenesis remain unclear and appear to be dependent on patients’ inflammatory status [33]. IL-6 also differentially affects osteoclast formation *via* soluble (*trans*) receptors but not membrane-bound (*cis*) receptors [34].

Very little is known about the peculiar phenotype of jaw bone cells in the context of FD. Because of differences in origin, functional constraints or clinical manifestations between the appendicular and cranio facial bones, it thus appears pivotal to better characterize skull-derived FD osteoblasts.

The aim of our study was to characterize primary cultures of human jaw osteoblasts from patients presenting fibrous dysplasia compared to healthy patients’ focusing on their proliferation and differentiation abilities, as well as their osteoclastogenic potential.

## Materials and methods

### Patients and tissues

Mandibular bone explant cultures were obtained during oral surgery from 7 patients presenting fibrous dysplasia (median age 28 (range 18–61); 2 males) and 8 non-FD patients (median age 18 (range 18–25); 4 males)(Supplementary Material 1). FD bone explants were obtained from resective lesional surgery and non-FD bone explants from wisdom tooth extraction. No ethical declaration was required for this study, bone samples were obtained after patients signed a written informed consent in accordance with the usual ethical legal regulations (Article R 1243-57) (CODECOH authorization DC-2014-2262) and according to the declaration of Helsinki. All FD and non-FD primary osteoblasts were obtained by migration from mandibular bone explants placed in in vitro culture, as described previously [35]. Fragments were cut into small pieces, washed in phosphate-buffered saline (DPBS, Gibco^®^, Thermo Fisher Scientific, Illkirch, France), three times for five min, digested in a solution of 0.5% trypsin-EDTA (Gibco^®^, Thermo Fisher Scientific, Illkirch, France) and then in 0.14% type II collagenase (Sigma Aldrich, Saint-Quentin Fallavier, France). The fragments obtained were placed in 25-cm² culture flasks containing 10 mL of DMEM (Gibco^®^, Thermo Fisher Scientific, Illkirch, France) supplemented with 20% of fetal bovine serum (Sigma Aldrich, Saint-Quentin Fallavier, France) and a 1% penicillin- streptomycin (Sigma Aldrich, Saint-Quentin Fallavier, France) solution, and incubated at 37 °C in a 5%-CO2 humidified atmosphere. When they reached confluency, cells were amplified in 75-cm² culture flasks (Falcon^®^, Dominique Dutscher, Burmath, France) with 10% of FBS-containing medium. The amplification step was repeated twice. In all experiments, cells were used at the third passage. FD and non-FD osteoblasts were cultured in the above-mentioned osteogenic differentiation medium to which were added 10 nM of dexamethasone [36], 10 mM of β-glycerophosphate and 50 mM of ascorbic acid [37] (both from Sigma Aldrich, Saint-Quentin Fallavier, France). Cells were seeded in 24-well plates (Falcon^®^, Dominique Dutscher, Burmath, France) for proliferation and on glass coverslips (Deckglaser, 12-mm diameter; Dominique Dutscher, Brumath, France) for stainings and immunostainings. A concentration of 2,500 cells per cm^2^ was seeded and covered with 1 mL of medium. Medium renewal was performed every 7 days. Supernatants were collected after 1, 7, 14 and 21 days, then stored at -20 °C. Cells were frozen at -80 °C, fixed according to the chosen procedure or used fresh to perform proliferation assays. 

### Histology

Mandibular bone explants were fixed in formaldehyde 4%. They were decalcified in formaldehyde 0.2% and EDTA 4.13%. Then samples were washed and dehydrated in a graded series of alcohol alcohol, and then alcohol was replaced by xylene. Samples were embedded in paraffin. Multiple serial sections of the samples were performed at 3-µm intervals.

After rehydration, sections were stained with hematoxylin and eosin or TRAP according to the manufacturer’s instructions (Sigma Aldrich, Saint-Quentin Fallavier, France).

All observations and acquisitions were performed using a Zeiss Axiovert 200 M microscope system (x 4 magnification, Carl Zeiss Axiovert 200 M, Axiovision Software, Marly le Roi, France).

### cAMP measurement

At 21 days of culture, cells were treated at 5 mM for 2 h of IBMX alone (a non-competitive phosphodiesterase inhibitor that increases intracellular cAMP) (Abcam, Cambridge, UK) or combined with 1 µM of forskolin (an activator of adenylate cyclase)(Abcam, Cambridge, UK) in order to be able to measure cAMP. Cells were then lysed in 300 µL of HCl solution (0.1 M) with 0.1% of Triton-X100 for 10 min at room temperature. Lysates were spun at 600 g for 10 min. cAMP concentration was measured with a cAMP ELISA kit (Enzo, Villeurbane, France).

### Proliferation assays

#### Mitochondrial activity

Mitochondrial activity was determined at days 1, 7, 14 and 21 using WST-1 assays (Cell Proliferation Reagent WST-1^®^, Roche, Saint-Quentin Fallavier, France) according to the manufacturer’s instructions. Absorbance was measured at 440 nm using a FLUOstar Omega microplate reader (BMG Labtech) against a background control being blank. A wavelength of 750 nm was used as correction.

#### DNA quantitation

DNA was isolated and purified using MasterPure™ DNA Purification kit (Epicentre, Madison, USA) in accordance with the manufacturer’s protocol. DNA was quantified by spectrophotometry (Nanodrop 2000 C, Thermoscientific). DNA purity was assessed by measuring the absorbance ratio at 260/280 which was comprised between 1.8 and 2.

### Osteoblastic phenotypic characterization

Markers of osteoblast differentiation and maturation (Alkaline Phosphatase (ALP) activity and extracellular matrix mineralization) were determined at days 7, 14 and 21.

#### Alkaline phosphatase staining

ALP staining was performed using *Sigma Fast BCIP/NBT* (Sigma Aldrich, Saint-Quentin Fallavier, France). Cells were fixed in 4% paraformaldehyde for 15 min. A tablet of *Sigma FAST BCIP/NBT* was diluted in 10 mL of distilled water. After washing, cells were stained with 300 µL of ‘ready-to-use’ solution for 15 min at room temperature protected from light. Reaction was stopped by washing with buffer. 1,200-ppp-resolution images were taken with a photographic scanner (EPSON Perfection 3200 Photo).

#### Alkaline phosphatase activity

ALP activity was measured using the p-nitrophenyl phosphate (pNPP) technique. Osteoblasts were lysed in an alkaline buffer (0.1 M Tris, 0.06% Triton X100 (both from Bio-Rad, Marnes-la-Coquette, France), 0.25 M NaCl, 0.02% NaN_3_ (both from Sigma Aldrich, Saint-Quentin Fallavier, France), pH 10.2). Cell lysate was incubated in a buffer containing 10 mM of aminomethyl propanol (Acros Organics, Thermo Fisher Scientific, Illkirch, France), 2 mM of MgCl_2_ (Merck, Fontenay-sous-bois, France) and 2 mM of pNPP (Fisher Bioreagents, Thermo Fisher Scientific^®^, Illkirch, France, pH 10.2), the latter used as a substrate, for 2 h at 37 °C. The ALP activity in the samples hydrolyses the pNPP, thus resulting in the production of inorganic phosphate and paranitrophenol (PNP) which is measured by spectrophotometry at 405 nm using FLUOstar Omega microplate reader (BMG Labtech, Champigny sur Marne, France) with a correction of non-specific background at 750 nm. ALP activity was simultaneously evaluated with a standard curve based on a serum containing known ALP activity. All results were normalized to the total amount of proteins contained in each well by using the BCA kit (Pierce BCA Protein Assay Kit - Thermo Fisher Scientific, Illkirch, France) according to the manufacturer’s instructions. Values were measured by spectrophotometry at 562 nm using a FLUOstar Omega microplate reader (BMG Labtech, Champigny sur Marne, France).

#### Alizarin red staining

For extracellular mineralization assays, osteoblasts were fixed in ice-cold ethanol for 1 h then washed with deionized water and stained with 2% alizarin red (Sigma-Aldrich, Saint-Quentin Fallavier, France) in deionized water for 1 h. Cells were washed twice with deionized water to eliminate non-specific staining. 1,200-ppp-resolution images were taken with a photographic scanner (EPSON Perfection 3200 Photo). Alizarin Red is used to highlight calcium deposit in extracellular matrix during osteoblastic differentiation [38].

#### Calcium ion concentration

To determine Ca^2+^ concentration, stained cells were destained using 1% cetylpyridinium chloride (CPC) monohydrate in 10 mM sodium phosphate (both from Sigma Aldrich, Saint-Quentin Fallavier, France) for 30 min at room temperature. Calcium ion concentration was evaluated with a standard curve based on a alizarin red solution containing known Ca^2+^ concentration. The optical density was read at 550 nm using a FLUOstar Omega microplate reader (BMG Labtech, Champigny sur Marne, France) with a correction set at 750 nm. All results were normalized to the total amount of proteins contained as previously described.

#### Type-I collagen and osteocalcin immunostaining

For type-I collagen and osteocalcin immunostaining, osteoblasts were cultured on glass cover slips. At confluency, cells were fixed for 20 min in 4% paraformaldehyde (Sigma Aldrich, Saint-Quentin Fallavier, France) with 0.15% picric acid added to it as requested by type-I collagen antibody manufacturer, then washed with DPBS. Cells were permeabilized with 3% BSA (Sigma Aldrich, Saint-Quentin Fallavier, France) plus 0.1% Triton X100 (Acros Organics, Thermo Fisher Scientific, Illkirch, France) for 45 min then incubated overnight at 4 °C with rabbit anti-human type-I collagen monoclonal antibody (clone H-197, Santa Cruz Biotechnology, Heidelberg, Germany) and mouse anti-human osteocalcin monoclonal antibody (clone 190125, R&D systems, Lille, France). Cells were rinsed in DPBS and blocked with 3% BSA for 30 min. After washing, cells were incubated with goat anti-rabbit biotinylated antibody (diluted 1/50 in 1% BSA) (Vector, Burlingame, California) for 30 min, rinsed in DPBS and incubated with a solution of streptavidine-Alexafluor^®^488 (diluted at 1/200, Molecular Probes, Thermo Fisher Scientific, Illkirch, France). After washing, cells were incubated goat anti-mouse antibody coupled with Alexafluor^®^568 (dilution at 1/50, clone AB_2534072, Invitrogen, Thermo Fisher Scientific, Illkirch, France). After rinsing, nuclei were counterstained for 5 min with DAPI (Invitrogen, Thermo Fisher Scientific, Illkirch, France). Glass coverslips were finally mounted (Fluorescent Mounting Medium, Dako, les Ulis, France). Controls were performed, consisting in the same procedure without the first antibodies, to assess non-specific background. Immunofluorescence images were obtained using a Zeiss Axiovert 200 M microscope system (x 40 magnification, Carl Zeiss Axiovert 200 M, Axiovision Software, Marly le Roi, France) and an axioCamMR3. All of the acquisitions were made with the same exposure time for the different channels (DAPI: 320 ms; FITC: 840 ms; Texas Red: 420 ms). Images were processed identically with ImageJ software (v1.50i, NIH, USA).

### Gene expression

For FD and non-FD osteoblasts at days 7, 14 and 21, total RNA were isolated and purified using MasterPure™ RNA Purification kit (Epicentre, Madison, USA) in accordance with the manufacturer’s protocol. RNA were quantified by spectrophotometry (Nanodrop 2000 C, Thermoscientific). RNA purity was assessed by measuring the absorbance ratio at 260/280, which was comprised between 1.8 and 2. Total RNA (250 ng) were reverse transcribed into cDNA using a High-Capacity cDNA Reverse Transcription kit (Applied Biosystems) following the manufacturer’s instructions. Using this approach, the transcriptional levels of: *RPS18 (*5’-3’ TGCGAGTACTCAACACCAACA; 3’-5’ GCATATCTTCGGCCCACA) and *GAPDH* (internal controls)*(*5’-3’ GAGTCCACTGGCGTCTTCAC; 3’-5’ GTTCACACCCATGACGAACA), *TNFRSF11B* (5’-3’ GAAGGGCGCTACCTTGAGAT; 3’-5’ GCAAACTGTATTTCGCTCTGG), *TNFSF11* (5’-3’ TGATTCATGTAGGAGAATTAAACAGG; 3’-5’ GATGTGCTGTGATCCAACGA), *IL-6* (5’-3’ GAAGGCAGCAGGCAACAC; 3’-5’ CAGGAGCCCAGCTATGAACT), *BGLAP* (5’-3’TGAGAGCCCTCACACTCCTC; 3’-5’ ACCTTTGCTGGACTCTGCAC) and *COL1A* (5’-3’ GGGATTCCCTGGACCTAAAG; 3’-5’ GGAACACCTCGCTCTCCAG). mRNA level of expression was determined using Power SYBR^®^ Green PCR Master MIX (Applied Biosystems). After a first denaturation step at 95 °C for 10 min, qRT-PCR reactions were performed according to a thermal profile that corresponded to 40 cycles of denaturation at 95 °C for 15 s, annealing and extension at 60 °C for 1 min. Data analysis was performed with StepOne™ Software v2.3 (Applied Biosystems). Data are expressed as target mRNA variation versus internal control RPS18.

### Osteoclastogenic potential

The concentration of IL-6 and OPG was assessed in FD and non-FD osteoblast culture supernatants, and total RANK-L in cell lysates. Concentrations were assessed using DuoSet^®^ ELISA kits (R&D systems, Lille, France) according to the manufacturer’s instructions at days 7, 14 and 21. All results were normalized to the total amount of proteins contained. For total RANK-L measurement, cells were lysed in alkaline buffer (50 mM Trizma-HCl; 10 mM NaF; 2 mM Na_3_VO_4;_ 2 mM PMSF; 1% Triton 100X, pH 8).

For membranous RANK-L immunostainings [35], 21-day confluent FD and non-FD osteoblasts were fixed in 4% paraformaldehyde for 15 min, without Triton X100, then washed twice with DPBS. Cells were blocked for 1 h in BSA 3% before incubation for 1 h with the mouse IgG2B anti-human RANK-L monoclonal antibody (1/50 diluted in BSA 1%, clone 70525, R&D Systems, Lille, France). After washing twice with DPBS, cells were blocked again with BSA 3% for 30 min. They were incubated for 30 min with the goat anti-mouse biotinylated antibody (diluted at 1/50 in BSA 1%, Vector). Cells were then rinsed in DPBS and incubated in a solution of streptavidin-AlexaFluor^®^488 (diluted at 1/200, Molecular Probes, Thermo Fisher Scientific, Illkirch, France) for 30 min. After rinsing, cells nuclei were counterstained for 5 min with DAPI (Invitrogen, Thermo Fisher Scientific, Illkirch, France). Glass coverslips were finally mounted (Fluorescent Mounting Medium, Dako, les Ulis, France). The control without the first antibody was performed to assess non-specific background. Immunofluorescent images were obtained using a Zeiss Axiovert 200 M microscope system (Carl Zeiss, Marly-le-Roi, France).

### Osteoclasts culture

#### Isolation of PBMCs and osteoclasts generation

Venous whole blood (12 ml) was drawn from six healthy volunteers (EFS Grand Est – ALC/PIL/DIR/AJR/FO/606) by venipuncture and stored in EDTA tubes (K2E, Becton Dickinson, Le Pont de Claix, France) for further processing. Peripheral Blood Mononuclear Cells (PBMCs) were purified using a density gradient with Polymorphprep™ (Proteogenix, Schiltigheim, France) according to our optimized differentiation protocol [39]. PBMCs were seeded on glass cover slips (Deckglaser, 12 mm of diameter; Dominique Dutscher, Brumath, France) into 24-well plates. A seeding density of 5 × 10^5^ monocytes per well was used for PBMCs. RAL staining (RAL 555, CellaVision, Mobilvägen, Swenden), a rapid variation of May-Grünwald Giemsa staining, was used according to manufacturer’s instructions to evaluated monocytes proportion. They were cultured in three different media: a control medium (RPMI medium 1640 Glutamax™ (Life Technologies, Courtaboeuf, France)/osteogenic differentiation medium (v/v)), a non-FD-conditioned medium (control medium/non-FD osteoblast supernatant (v/v)) and a FD-conditioned medium (control medium/FD osteoblast supernatant (v/v)). Conditioned media were respectively obtained from FD (*n* = 7) and non-FD (*n* = 8) osteoblast cultures which were performed in 75 cm^2^ culture flasks at a 2,500-cell-per-cm^2^ density and using 10mL of the osteogenic differentiation medium previously described. At 21 days of culture, supernatants (7-day accumulation) were collected and stored at -80 °C. OPG concentration was controlled by the above-mentioned ELISA test. An average concentration of 3,500 pg/mL was measured in FD-conditioned supernatants and 2,000 pg/mL in non-FD-conditioned medium.

All media were supplemented with 10% of fetal bovine charcoal-stripped serum (v/v) (Sigma Aldrich, Saint- Quentin Fallavier, France), 25 ng.ml^− 1^ of recombinant human M-CSF and 50 ng.ml^− 1^ of recombinant human RANK-L (Miltenyi Biotec SAS, Mercoeur, France) for 21 days at 37 °C, in a humidified atmosphere containing 5% of CO_2_ all along. The medium was removed and replaced with fresh medium every 48–72 h.

#### Osteoclasts characterization

Supernatants were removed after day 21 and cells were fixed with 4% paraformaldehyde (v/v in PBS without Ca^2+^ and Mg^2+^) (Sigma Aldrich, Saint- Quentin Fallavier, France) for 10 min at 37 ◦C, then permeabilized with Triton X100 0.5% (v/v in PBS without Ca^2+^ and Mg^2+^) (Sigma Aldrich, Saint- Quentin Fallavier, France) for 15 min at room temperature. After two 5-min washes in PBS (without Ca^2+^ and Mg^2+^), actin cytoskeleton was stained with AlexaFluor^®^488-conjugated Phalloidin (1/100 v: v) (Invitrogen) diluted into BSA 0.5% (v/v in PBS without Ca^2+^ and Mg^2+^) for 30 min at room temperature and protected from light. Cells were then washed twice with PBS without Ca^2+^ and Mg^2+^ for 5 min. The nuclei were labeled with DAPI 1/3,000 (v/v in distilled water) for 5 min at room temperature protected from light, then washed twice with distilled water. Cells were visualized by fluorescent microscopy. Each analysis was performed on 5 randomly chosen microscope fields for each well (×20 magnification, Zeiss Axiovert 200 M, Axiovision Software, Zeiss, Marly-le-Roi, France). Images were processed with ImageJ software (v1.50i, NIH, USA). Cell counter plugin was used to count multinucleated OCs and their nuclei. Actin ring positive cells bearing 3 or more nuclei were considered to be OCs. For the area measurement, we used a freehand selection tool. At least 5 OCs were analyzed per field for each well when counting nuclei and measuring areas.

### Statistical analysis

DNA and metabolic activity data were expressed as median value +/- first and third quartiles. All other data were expressed as whisker plots (the red bar representing median value, the upper and lower end of the box the first and third quartiles and the upper and lower bars the first and ninth decile).

Statistical differences were calculated by non-parametric Kruskal-Wallis test followed *by post hoc* exact and stratified (when relevant) Wilcoxon-Mann-Whitney test (StatXact 7.0, Cytel Inc, Cambridge, MD, USA). *p* < 0.05 was considered significant.

## Results

### FD mandibular bone presents classical features

**Fig. 1:**
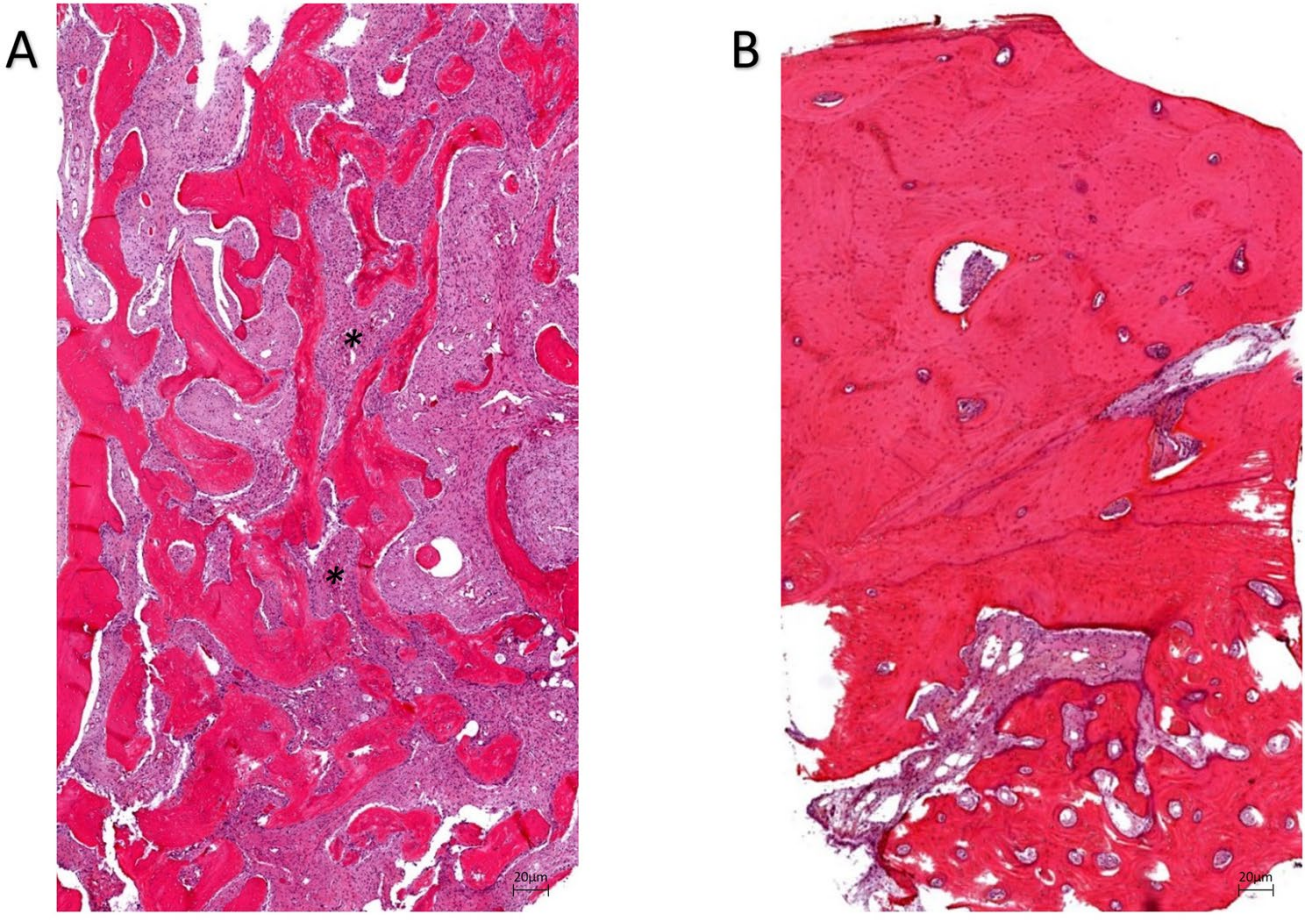
Representative non-FD bone sections (**A**) present a large mineralized matrix whereas representative FD lesions (**B**) are composed of fibrous tissues between bone trabeculae. FD trabeculae appear non-stress oriented and disorganized. In FD sections, a typical ‘alphabet soup’ (*) is caused by disorganization of woven and lamellar bone. Images were acquired with a Zeiss Axiovert 200 M inverted microscope. Scale bar: 20 μm

Bone trabeculae in FD lesions presented characteristic spicules of woven bone composed of collagen fibrils (Fig. 1.B). FD lesions consist of dysplastic and disorganized fibrous tissues. FD biopsies are marked by a typical “alphabet soup” appearance [40, 41] caused by the tangled arrangement of woven and lamellar bone tissue. In contrast, non-FD bone tissue is characterized by an organized lamellar-collagen-fiber and a heterogeneous lamellar bone structure (Fig. 1.A).

### FD osteoblasts from mandibular explant are more proliferative

iferative

**Fig. 2:**
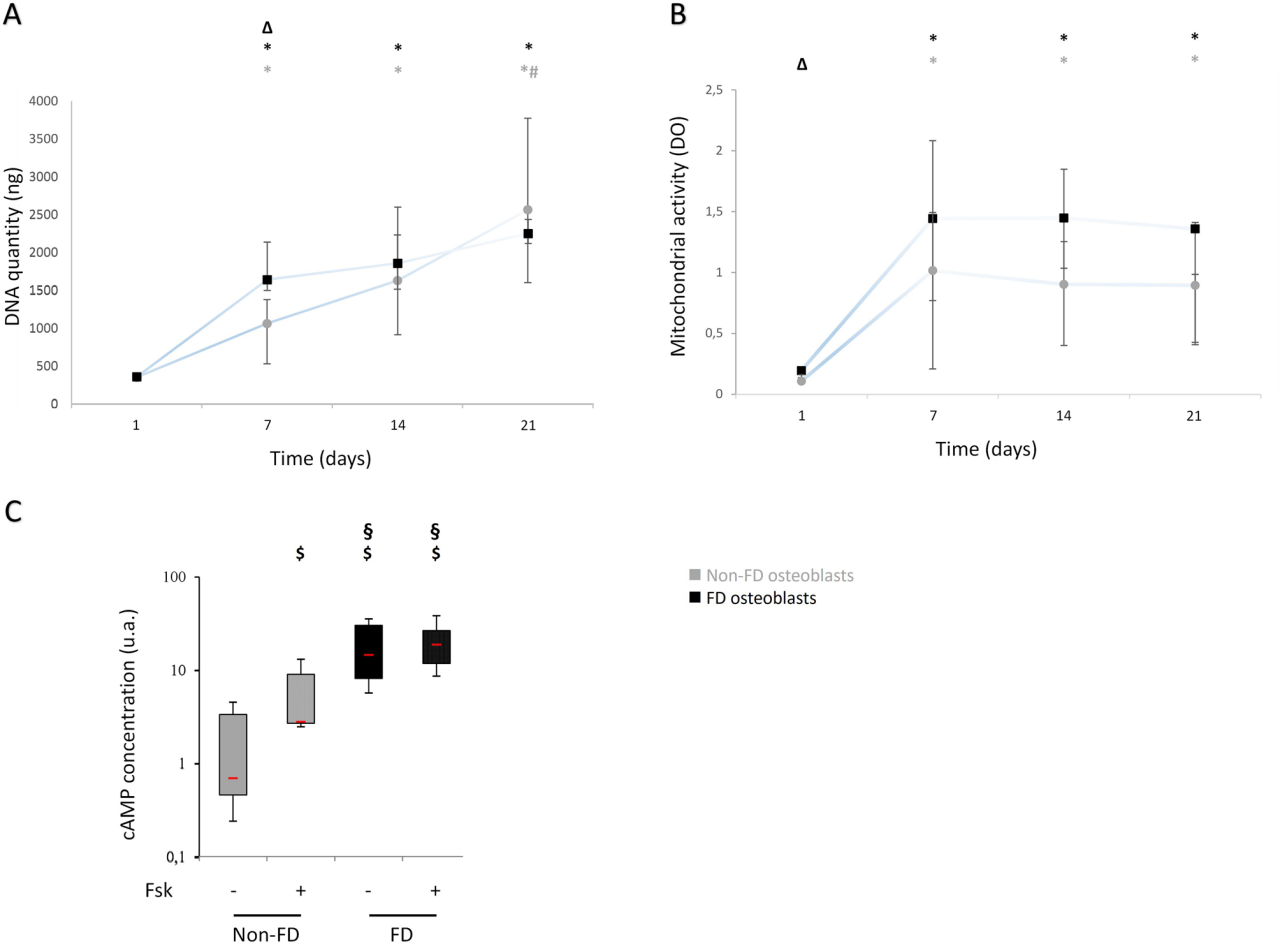
Characteristics of FD osteoblasts culture. FD osteoblasts are more proliferative than non-FD osteoblasts. DNA quantity (**A**) and mitochondrial activity (**B**). Grey circles represent non-FD osteoblasts and black squares represent FD osteoblasts. (**C**) cAMP is increased in FD culture compared to non-FD culture in IBMX alone and IBMX + Fsk conditions. No variation is observed with Fsk in FD cultures. Grey values represent non-FD cell and black values represent FD osteoblasts. FD, *n* = 7; non-FD, *n* = 8. $ *p* < 0.05 versus non-FD_IBMX; § *p <* 0.05 FD versus non-FD_IBMX + Fsk; Δ *p <* 0.05 versus non-FD; * *p* < 0.05 versus J1; # *p* < 0.05 versus J7

Between day 1 and day 7, DNA quantity in FD osteoblasts increased and then reached a plateau. In non-FD culture, an increase in DNA quantity was observed between day 1 to day 14, reaching a plateau between day 14 and day 21. Interestingly, at day 7, FD osteoblasts exhibited a 53% higher DNA quantity compared to non-FD osteoblasts. From day 14, DNA quantities were equal in both cultures (Fig. 2.A). FD osteoblasts’ doubling time was twice as short as non-FD osteoblasts’ between day 1 and day 7 (respectively, 1.15 days *versus* 2.59 days). WST-1 analysis in FD and non-FD cultures evidenced a similar pattern. Metabolic activities increased between day 1 and 7, then reached a stationary phase until day 21 (Fig. 2.B). Metabolic activity in FD culture was higher at the first time point compared to non-FD culture.

*GNAS* mutations result in elevated intracellular cAMP concentration, which is a hallmark of FD osteoblasts with constitutive overactivation of Gαs. At day 21 of culture, under basal IBMX condition, FD osteoblasts exhibited a higher intracellular cAMP concentration compared to non-FD osteoblasts. As expected, adenylyl-cyclase activation by Forskolin significantly increased cAMP in non-FD osteoblasts (+ 384%, *p =* 0.01). In FD culture, no significant variation was observed with adenylyl-cyclase activator (*p* = 0.08). However, in IBMX+Forskolin conditions, cAMP remained higher in FD cultures compared to non-FD cultures. Even with activation of cAMP production, intracellular concentrations in non-FD osteoblasts did not reach those measured in basal FD cultures (*p =* 0.01 between non-FD_IBMX+Fsk_ and FD_IBMX_)(Fig. 2.C).

FD osteoblasts exhibit non-fully differentiated phenotype.

**Fig. 3:**
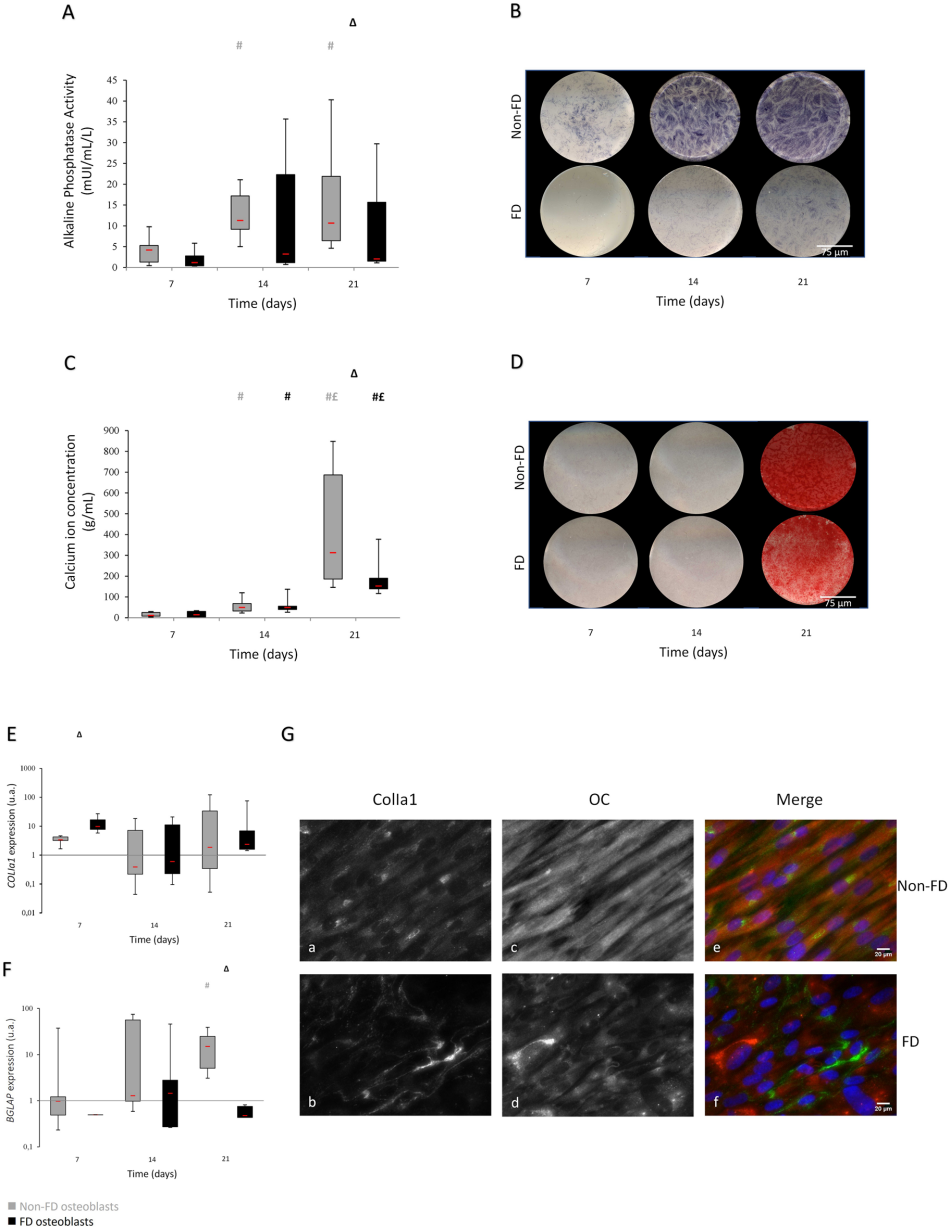
FD osteoblasts exhibit defective phenotype. ALP quantification (**A**) and staining (**B**). Calcium concentration (**C**) and Alizarin Red staining (**D**). Scale bar: 75 μm (**B**, **D**); COL1A1 (**E**) and BGLAP (**F**) gene expression measurement by RT-qPCR. (**G**) Representative photographs of FD and non-FD cell cultures exhibit different type-I collagen and osteocalcin expression at D21 of culture. Type-I collagen (a, b) and Osteocalcin (c, d) immunostainings. Colorized overlayed images (e, f): ColIa1 (AlexaFluor488)/OC (AlexaFluor568)/nucleus (DAPI). Grey values represent non-FD osteoblasts and black values represent FD osteoblasts. Images were acquired with a Zeiss Axiovert 200 M inverted microscope. Scale bar: 20 μm (G: a,b, c,d, e,f). FD, *n* = 7; non-FD, *n* = 8. Δ *p <* 0.05 FD versus non-FD; # *p <* 0.05 versus D7; £ *p <* 0.05 versus D14

FD osteoblasts presented consistently low and stable ALP activity at all time points, whereas non-FD osteoblasts presented an increased activity between day 7 and day 14 which then stabilized between day 14 and day 21. At day 21, FD osteoblasts displayed a statistically significant decrease in ALP activity compared to non-FD osteoblasts (-80%, FD versus non-FD, *p* = 0.02) (Fig. 3.A). ALP stainings reinforced these observations; FD osteoblasts exhibited a lower staining than non-FD osteoblasts without any visible evolution throughout time (Fig. 3.B).

Alizarin red staining was very low in both conditions, at day 7 and 14 (Fig. 3.C-D). Clear alizarin red stainings were only clearly detectable at day 21 in both FD and non-FD cultures (Fig. 3.D). At day 21, a significant difference in calcium concentration could be seen between FD and non-FD osteoblasts. The staining in FD osteoblasts was less intense and homogeneous than non-FD osteoblasts.

COL1A1 gene expression was significantly increased at day 7 in FD osteoblasts compared to non-FD osteoblasts. At day 14 and 21, expressions levels were equivalent in both conditions (Fig. 3.E). *BGLAP* was weakly expressed in both FD and non-FD cultures at day 7 and day 14. However, its expression was significantly increased in non-FD osteoblasts at day 21 compared to FD osteoblasts. While *BGLAP* expression was stable over time in FD cultures, it was significantly increased between day 7 and day 21 in non-FD osteoblasts (Fig. 3.F). At 21 days culture, all non-FD osteoblasts expressed intracellular type-I collagen and osteocalcin (Fig. 3.G: a,c, e). These immunostainings were intense and uniform in all cells. In comparison, immunostainings were weaker and more heterogeneous in FD culture (Fig. 3.G: b,d, f). The intensity of intracellular type-I collagen varied among FD osteoblasts, and an intense extracellular fluorescent signal was detected, interestingly displaying the presence of extracellular collagen fibrils. Osteocalcin immunostaining was less intense and detected in fewer cells in FD culture (Fig. 3.G: c,f).

### Osteoclastogenesis potential is reduced in FD cultures and in histological bone section

**Fig. 4:**
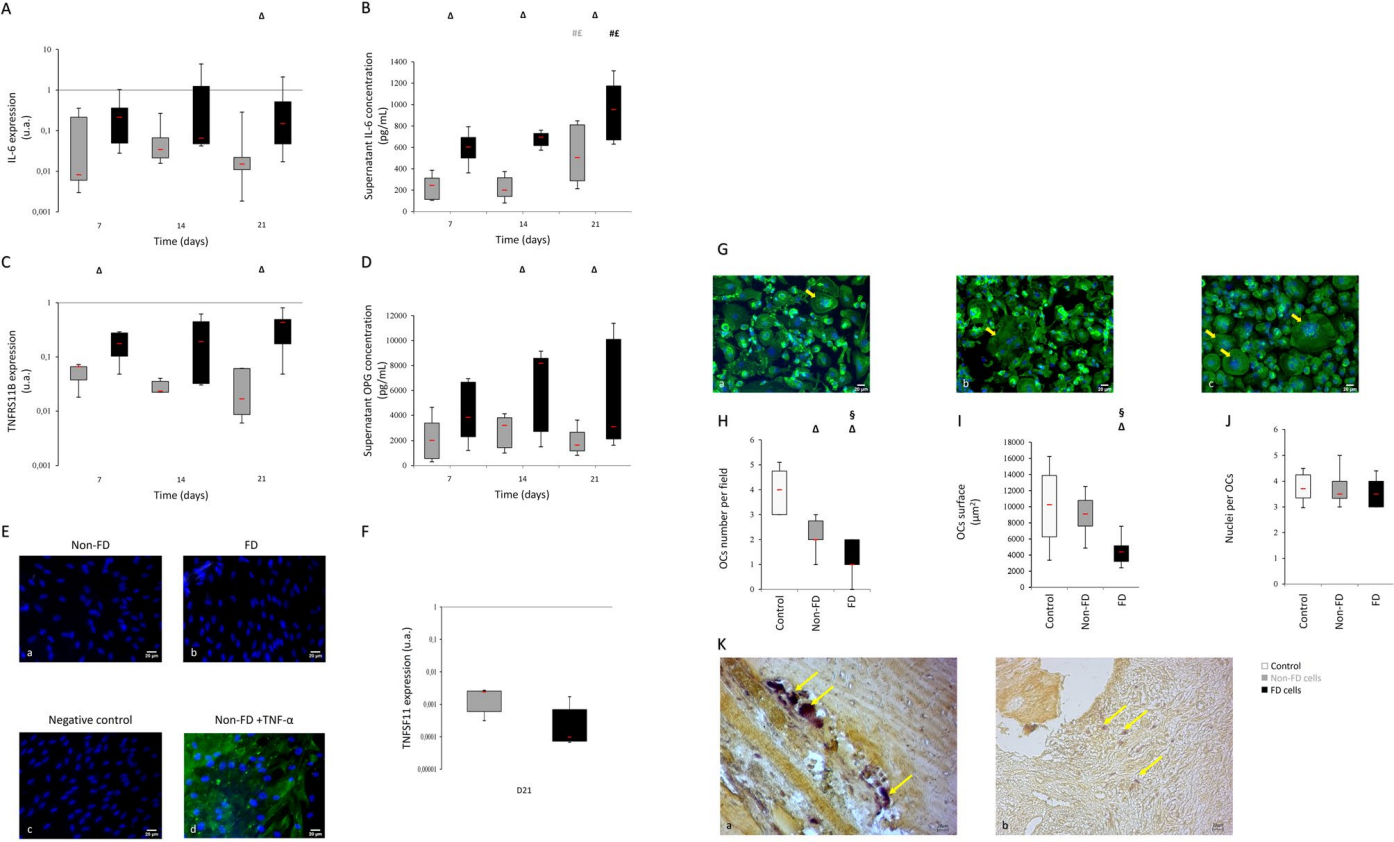
Osteoclastogenesis potential is decreased in FD osteoblasts culture and in bone. A higher IL6 and OPG secretion are observed in FD cell culture measured by RT-qPCR (**A**-**C**) and ELISA (**B**-**D**). RANK-L expression couldn’t be detected by immunochemistry (**E**) at D21 in non-FD culture (**E**: a) or in FD culture (**E**: b). RANK-L immunostaining negative control (**E**: c) and a positive control under TNF-α stimulation (20ng/mL)(**E**: d). Colorized overlayed images: RANK-L (AlexaFluor^®^488)/nucleus (DAPI). Scale bar: 20 μm. RANK-L expression was detectable only at D21 by RT-qPCR (**F**). (**G**) Representative photographs of F-actin medium (**G**: a), non-FD medium (**G**: b) and control (**G**: c) cultures. Colorized overlayed images: Phalloidin-AlexaFluor^®^488/nucleus (DAPI). Scale bar: 20 μm. A decreased OCs number in FD-medium culture is observed compared to non-FD-medium culture and control medium (**H**). FD medium induced a decreased OCs surface (I). No variation in nuclei number per OCs between FD and non-FD cultures (**J**). Representative non-FD bone sections (**K**; a) presented a higher TRAP staining compared to FD bone section (**K**; b). White values represent control, grey values represent non-FD osteoblasts and black values represent FD osteoblasts. FD, *n* = 7; non-FD, *n* = 8 Control medium, *n* = 6; FD-medium, *n* = 6; non-FD-medium, *n* = 6. Δ *p <* 0.05 versus control; § *p <* 0.05 versus non-FD

Analysis of IL-6 mRNA showed a stable gene expression throughout time in both cultures (Fig. 4.A). IL-6 gene expression in FD osteoblasts was higher than non-FD osteoblasts’ with a statistically increased expression observed at day 21 (*p =* 0.002 FD *versus* non-FD). At the protein level, IL-6 secretion in both cell cultures exhibited no time-dependent variation until day 14. An increase in IL-6 production was observed at day 21 in both FD and non-FD osteoblasts. IL-6 concentration was consistently higher in FD osteoblasts compared to non-FD osteoblasts (Fig. 4.B).

On the one hand, RANK-L protein was below our detection levels either by ELISA or by immunostaining, regardless of the culture duration. *TNFSF11* gene expression was detected only at day 21 in both cultures and appeared to be higher in non-FD osteoblasts than in FD without significant difference (Fig. 4.F). On the other hand, OPG gene (*TNFRSF11B*) expression and protein secretion were observed in both cultures (Fig. 4.C). At all times, OPG expression in FD osteoblasts was higher than non-FD’s (Fig. 4.D). These increases were statistically significant at day 7 and day 21. No variation throughout time was observed in both conditions. OPG protein measurement revealed a statistically significant increase in secretion at day 14 and day 21 in FD cell cultures compared to non-FD (Fig. 4.D). As for *TNFRSF11B* mRNA expression, no variation was observed throughout time at protein level.

At day 21, PBMCs were twice as engaged in osteoclastogenesis when cultured in non FD-conditioned medium (Fig. 4.G; a) as when cultured in FD-conditioned medium (Fig. 4.). OCs in control medium (Fig. 4.C) were more numerous than those in both conditioned media (Fig. 4.D). No variation in OCs surface was observed between control medium and non-FD-conditioned medium. However, OCs cultured in FD-condition were smaller (Fig. 4.E). No significant difference in nuclei number per OCs was found between the control culture, FD-conditioned culture (3.5 nuclei number per OCs, median value) and non-FD-conditioned medium (Fig. 4.F).

In bone section from FD patients, TRAP positive cells were fewer compared to non-FD patients (Fig. 4.K). Also, osteoclasts seemed to be smaller in FD patients.

## Discussion

Mandibular bone biopsies from FD patients presented FD lesions’ typical ‘alphabet soup’ characterictics [40, 41], confirming the diagnosis of fibrous dysplasia [42]. *GNAS* mutations causing fibrous dysplasia are also known to increase cAMP in FD osteoblasts from several tissues including appendicular bones [4, 43] and the same results were observed in osteoblasts from cranio-facial lesions. Indeed, cAMP measurement in FD osteoblasts were higher than non-FD osteoblasts’. Even if cAMP concentration increased in non-FD cultures under forskolin stimulation, its synthesis remained lower compared to that of FD osteoblasts with or without forskolin stimulation. Interestingly, we observed no variation in forskolin stimulated FD osteoblasts’ cAMP concentrations compared to unstimulated ones. These results suggest that cAMP production in FD osteoblasts from mandibular lesions is maximal and constitutive.

Jaw bone cells from FD patients exhibited increased proliferation compared to cells from non-FD patients. Cell proliferation was assessed using a combination of DNA and WST-1 assays, methods often employed in combination to evaluate cell proliferation and viability [44, 45]. These results are consistent with a previous study conducted by Marie and collaborators showing a proliferation twice as high in FD cultures from frontal bone at first and third day of culture [4]. In our study, even if we used a longer culture time (up to day 21), FD osteoblasts presented a higher proliferative capacity only seen at the first time point. This is probably due to the limited area of the culture wells that preclude visible differences at later time points, as once they have fully colonized the available substrate, cells stop their proliferation. FD is caused by a *GNAS* variants that increases cAMP. This augmentation could increase amphiregulin secretion [46] or *c-fos* proto-oncogen [23, 47] in FD lesions and be responsible for increased proliferative capacities. FD osteoblasts also exhibit higher metabolic activity. The latter was particularly interesting because such an increased metabolic activity may have a clinical impact, especially when making a diagnosis. Indeed, bone scintigraphy is used to perform positive diagnosis and disease mapping. The degree of the isotope fixation was a marker of the bone metabolic activity [48]. Apart from their proliferative abilities, FD osteoblasts from the appendicular skeleton were characterized by a decrease in osteoblastic differentiation as demonstrated by histological studies showing the presence of immature and extensive osteoid tissue as well as extensive fibrosis [23]. In our experiments, ALP staining and quantification data, combined with type I collagen and osteocalcin immunostaining, highlighted the same features in jawbone cells differentiation in vitro. We observed decreased expression of intracellular type-I collagen and ALP activity, both of them early markers of osteoblastic differentiation. We detected the presence of extracellular type-I collagen in FD cell cultures. As collagens are the main components involved in fibrosis, we think that type-I collagen secretion observed in our conditions could echo fibrosis formation in FD lesions. Other studies observed that decreased ALP immunostaining was associated with a decreased osteocalcin staining in immunohistochemistry analysis [24, 46, 49].

These observations were in accordance with our results showing a lack of osteocalcin expression, a late marker of osteoblastic differentiation. At the same time, our data suggested a decrease in mineralization in FD culture. Indeed, calcium ion concentration in FD cultures was twice as low as non-FD osteoblasts. These observations indicated that the osteoid matrix present in FD lesions was the consequence of the osteoblast differentiation deficiency, causing a decreased mineralization rate.

Cell differentiation and proliferation present an interesting inverse relationship. Indeed, precursor cells proliferate until they acquire specialized functions leading to a complete differentiated state. This switch represents a crucial step in tissue formation [50]. In our conditions, even if FD osteoblasts present a higher proliferative rate leading to higher expression of *COLIA1 (*an early marker of osteoblastic differentiation), FD osteoblasts seemed not to efficiently engage in the complete osteoblastic differentiation process.

In our culture, fibrous FD osteoblasts showed an increased IL-6 production. These results are consistent with the finding of Yamamoto and collaborators’ work that highlighted an increased IL-6 synthesis in osteoblast cultures from MAS patients [28, 51, 52].

Interestingly, they showed that dibutyryl cAMP supplementation only improved IL-6 production in normal cells but not in FD osteoblasts. This suggests that FD osteoblasts perform a maximal IL-6 production. IL-6 expression is mediated by Gs-alpha, that is overactivated in FD [51]. Various studies in the literature described the stimulating effect of IL-6 on osteoclastogenesis and bone resorption [31, 52] and it is known that inflammatory conditions impair bone homeostasis [53]. In mouse model, IL-6’s role in osteoclastogenesis is bidirectional. Regulation depends on local RANK-L concentration. Feng et al. showed that the IL-6/sIL-6R complex significantly promotes osteoclast differentiation at low RANK-L levels (10 ng/ml), while it inhibits differentiation at higher levels (50 ng/ml) [54]. These findings suggest that IL-6 contributes to the dysregulated bone remodeling in FD by modulating osteoclastogenesis in a context-dependent manner [55].

However, a pro-osteoblastogenesis effect of IL-6 has been highlighted through IL-6R modulation [32, 56]. In addition, IL-6 didn’t have a direct action on osteoclast progenitors and osteoclastogenesis [34] but stimulated RANK-L expression in osteoblasts [57].

Studies have demonstrated that IL-6 can directly inhibit osteoclast differentiation under certain conditions. Yoshitake and colleagues showed that IL-6 specifically suppresses RANK-mediated IκB degradation and JNK activation, and that this suppression involves transcriptional regulation of specific genes related to MAPK phosphatases (MKP1 and MKP7) and the ubiquitin pathway (Senp2 and Cul4A) [32]. This direct inhibitory action of IL-6 suggests that this cytokine may play a dual role in regulating osteoclastogenesis, acting as a pro-resorptive factor in some contexts (notably through the induction of RANK-L in osteoblasts) and as a bone protector in others (by direct inhibition of osteoclast precursors) [55].

Regarding IL-6 rise in FD patients, anti-IL-6R treatment has been proposed to reduced bone pain and regulate bone resorption in patients presenting fibrous dysplasia [58]. However, no difference in CTX and other bone resorption biomarkers has been showed in patients treated with Tocilizumab, a monoclonal antibody targeting the IL-6 receptor. IL-6 is expected to have an indirect pro-osteoclastogenesis effect through osteoblasts activities, especially when RANK-L is low [52]. But immaturity of FD osteoblasts may preclude such an osteoclastogenesis stimulation and may account for Tocilizumab treatment failure and bone homeostasis imbalance.

The literature also reported an osteoclastogenesis stimulation on iliac FD lesions. An increased osteoclast number and TRAP activity has been shown in an histological study on iliac explants [59]. Recently, de Castro and collaborators showed a lower OPG/RANK-L ratio by increasing RANK-L and decreasing OPG secretion in iliac osteoblast cultures [60]. In contrast to previous findings, RANK-L remained under detection thresholds in our hands at protein level, and we evidenced an increased OPG expression and secretion in FD osteoblasts. De Castro and collaborators detected RANK-L secretion only in conditioned media, indeed cells were pre-treated for 48 h with prostaglandin E_2_ and 1,25 vitamin D_3_. However, it seems that they were able to detect RANK-L in cell lysates in FD and non-FD with and without stimulation media [60]. RANK-L acts as a pro-osteoclastogenic mediator through its interaction with its RANK receptor, a transmembrane receptor in osteoclast precursors [61, 62]. OPG is a soluble receptor of RANK-L, which is able to block RANK-L/RANK interaction and inhibit osteoclast differentiation [62]. Currently, denosumab, an anti-RANK-L therapy, has been proposed to treat FD lesions sustained by in vitro studies [63, 64]. Recently, a French retrospective study conducted in 6 rheumatology academic centers highlighted a good tolerance of denosumab in 13 FD/MAS patients, associated with a decreased pain evaluation [65]. Clinical studies [21, 66, 67] have shown a decreased in bone turnover markers with denosumab treatment leading to a promising therapeutics in treatment. Interestingly, de Castro and collaborators [68] highlighted that denosumab decreased the expression of GNAS variants in FD lesions. These results could reinforce the effects of denosumab in FD treatment. Nevertheless, authors recommend caution in the absence of randomized clinical studies [6, 69]. In our condition, OPG/RANK-L ratio couldn’t be evaluated. However, our data indicate a decreased osteoclastogenic potential in FD culture associated to an increased OPG secretion. Our results seemed to highlight a lack of osteoclastogenic differentiation in osteoclast cultures subjected to conditioned FD media. To further investigate the role of osteoclasts in bone remodeling in the context of FD, we examined osteoclastogenesis and found that PBMCs cultured in FD-conditioned medium presented a reduced osteoclastic differentiation with less osteoclasts of reduced surface. The decreased osteoclastogenesis may be attributed to a higher concentration in OPG. In FD lesions, osteoblasts were immature, and it seemed that RANK-L expression and secretion was altered, decreasing osteoclast differentiation. The impact on osteoclast resorptive activity remain to be determined to fully embrace the importance of these paracrine modulation in cranio-facial FD progression. Nevertheless, these observations were supported by TRAP staining performed on FD and non-FD histological bone sections that evidenced a decreased staining in lesional bone. Further experiments dedicated to osteoclasts biology are needed to explore the hypothesis of reduced osteoclastogenesis in FD mandibular lesions.

These distinctions between craniofacial and appendicular lesions could be explain by the embryologic and developmental origins of these two sites. On the one hand, craniofacial bones derive from cranial neural crest cells and develop through intramembranous ossification [70]. On the other hand, appendicular bones derivate from lateral plate mesoderm and ossification pathways occurred through endochondral process [71]. These variations are maintained in skeletal stem cells (SSCs). Intramembranous ossification is mediated by IFITM5 + craniofacial stem cells. Intramembranous ossification may inherently involve reduced osteoclastic coupling compared to endochondral ossification mediated by appendicular stem cells CADM1 + in long bones. Endochondral ossification necessarily involves extensive bone resorption of cartilage templates and trabecular remodeling, potentially creating a greater stimulus for osteoclastogenesis [72]. Conversely, intramembranous ossification in craniofacial sites may rely more heavily on osteoblast maturation and direct bone formation with reduced osteoclastic participation in the physiologic process.

An additional biological distinction between craniofacial and appendicular sites involves the local microenvironment. Craniofacial bones, particularly the mandible, possess rich vascularization with higher rates of bone marrow stromal cell proliferation compared to appendicular sites such as the ilium [73]. This enhanced vascularity in craniofacial tissues may contribute to differential immune cell trafficking, factor secretion patterns, and cellular heterogeneity within FD lesions.

The use of primary human bone cells in this study may have contributed to the observed heterogeneity in the results, which is a common characteristic of monolayer cell cultures [74–76].

Our results suggested that a difference may arise thanks to osteoblasts secreted factors between appendicular and cranio-facial FD lesions regarding osteoclastogenesis potential. Such a difference could explain radiographic divergence as far as localization is concerned. Radiolucent lesions appeared in appendicular skeletons, presenting an increased fracture risk. Radiopaque, dense and sclerotic lesions characterize hypertrophic cranio-facial lesions [77, 78]. These original data also interrogate about pharmacological prescriptions in cranio-facial lesions management. Indeed, bisphosphonates (BPs) are anti-resorptive agents primarily prescribed to manage osteoporosis, osteopenia, cancer-related conditions or skeletal-related events associated with metastases [79]. By inhibiting mevalonate pathways, BPs decreased osteoclast differentiation and bone resorption [80]. As appendicular skeleton presented an increased risk fracture and a low bone density, BPs were proposed in order to limit the loss of bone density and fracture or to reduce bone pain [81]. However, no or low effect on pain and radiographic appearance was reported [20, 22]. Moreover, Medication-Related Osteonecrosis of the Jaw (MRONJ), an established side effect of anti-resorptive therapy [79], has been described in patients presenting with FD [82, 83]. Whereas MRONJ prevalence is rare and evaluated between 0.04 and 0.1% in an osteoporotic population treated by BPs [79], 3,4% (12 out of 312 patients) to 5.4% (4 out of 76 patients) of FD patients treated by BPs developed MRONJ (3 FD-affected areas and 1 in non-FD-affected area) [82, 84]. These increased prevalence of MRONJ in FD population could be explained by the decreased osteoclastogenic potential in FD lesions, aggravated by the BPs effects that increase OPG/RANK-L ratio. Otherwise, anti-resorptive therapies could also exacerbate cranio-facial lesions. The variations observed between cranio-facial and appendicular bones could be explained by several differences, notably concerning their embryological origin and ossification processes: axial skeleton derives from mesoderm and exhibits endochondral bone formation [85] whereas bones from maxillofacial area (like jaw or teeth sockets) originate from neural crest inducing intramembranous bone formation process [86]. These differences lead to a difference in gene expression between the two sites [87, 88] and a distinct phenotype in marrow stromal cells [89–91]. A variation was detected in the function and mechanical forces applied on the bones according to skeletal site. Moreover, jaw bones (mandible and maxilla) development and structure are influenced by tooth eruption, movement, agenesis and extraction. Finally, multiple factors could be observed influencing bone metabolism such as sexual hormones, parathyroid hormone or antiresorptive drugs depending on location [92–95]. In fibrous dysplasia studies, Hopkins et al. recently reviewed different animal models used for pre-clinical fibrous dysplasia studies. Interestingly, no model evidenced a mandibular or maxillary bone expression of the mutation, although not all features were highlighted in a single model [96]. Even if our study presented a small number of donors because of scarcity of explants, our results seemed to argue that a specific treatment is needed to manage cranio-facial lesions in fibrous dysplasia focusing on osteoblast maturation and not solely on osteoclast differentiation.

## Conclusion

Our findings demonstrate that craniofacial FD lesions share similar characteristics with appendicular lesions, including increased cAMP concentration, high proliferative capacity, and defective osteoblastic phenotype. However, unlike appendicular FD lesions, craniofacial FD lesions exhibit decreased osteoclastogenic potential, as evidenced by increased OPG and IL-6 production. This difference may contribute to the clinical and radiological variations observed between appendicular and craniofacial FD lesions. Our results highlight the importance of investigating craniofacial FD lesions using human bone samples to better understand the pathophysiology of FD in this specific skeletal region.

## Supplementary Information

Below is the link to the electronic supplementary material.

Supplementary Material 1

## Data Availability

Data will be made available on request. The raw data supporting the conclusion of this article will be made available by the authors, without undue reservation.
