# Supplementary material for: In vitro characterization of osteoblasts from craniofacial fibrous dysplasia of bone and their impact on bone homeostasis
Source: Orphanet J Rare Dis. 2026 Feb 24;21:115. doi: 10.1186/s13023-026-04262-0 (PMC13041429; doi:10.1186/s13023-026-04262-0)
Supplement: Supplementary file 1 — Supplementary Material 1 [file 13023_2026_4262_MOESM1_ESM.docx]

**Supplementary material 1. Demographic and Clinical Characteristics of Fibrous Dysplasia and Control Donors**

| **Donor** | **Age (years)** | **Gender** |
| --- | --- | --- |
| FD1 | 19 | Female |
| FD2 | 31 | Male |
| FD3 | 28 | Female |
| FD4 | 18 | Female |
| FD5 | 18 | Female |
| FD6 | 23 | Male |
| FD7 | 61 | Female |
| Healthy 1 | 25 | Male |
| Healthy 2 | 18 | Male |
| Healthy 3 | 18 | Female |
| Healthy 4 | 18 | Female |
| Healthy 5 | 19 | Female |
| Healthy 6 | 18 | Male |
| Healthy 7 | 18 | Male |
| Healthy8 | 18 | Female |
